# Genetic diversity and population structure of the indigenous goat population in Tunisia's northwest

**DOI:** 10.5194/aab-68-409-2025

**Published:** 2025-06-20

**Authors:** Ikram BenSouf, Ines Dhib, Safa Bejaoui, Hatem Ouled Ahmed, Hichem Khemiri, Naceur M'Hamdi

**Affiliations:** 1 Laboratory of Animal, Genetic and Feed Resources (LRGAA), National Agronomic Institute of Tunisia, University of Carthage, 43 Avenue Charles Nicolle, Tunis, 1082, Tunisia; 2 Département des sols et de génie agroalimentaire, Faculté des sciences de l'agriculture et de l'alimentation, Université Laval. 2425, rue de l'Agriculture, Québec, QC, G1V 0A6, Canada; 3 Research Unit of Biodiversity and Resource Development in Mountain Areas of Tunisia, UR17AGR14, Higher School of Agriculture of Mateur, Carthage University, Tunis, Tunisia; 4 Animal Production and Health Laboratory, Joint FAO/IAEA Centre for Nuclear Applications in Food and Agriculture, Department of Nuclear Sciences and Applications, International Atomic Energy Agency, Friedenstrasse 1, 2444 Seibersdorf, Austria; 5 North West Sylvo-pastoral Development Office, Avenue de l'Environnement, 9000, Béja, Tunisia

## Abstract

Elucidating genetic diversity is critical for improving breeding strategies. To this end, the present study investigated the genetic diversity and population structure of the local goat population sampled from the northwestern region of Tunisia. The genetic variability was estimated using seven microsatellites, which revealed high diversity and genetic population clustering with a dispersed geographical distribution. All of the markers showed a significant genetic polymorphism, with an average of 18 alleles. Thus, 123 alleles were detected in the seven loci of the six studied populations. This result reflects the existence of a significant polymorphism within the local goat population. Overall, the highest $H_{O}$ was 0.952, and the highest $H_{E}$ was 0.942. Furthermore, the goat population demonstrated negative $F_{\mathrm{IS}}$ ( -0.177 ). This negative value shows an overall excess of heterozygotes, suggesting the absence of inbreeding. Analysis of molecular variance (AMOVA) revealed that genetic differences within the population explained 92.4 % of the variation. A low average $F_{\mathrm{ST}}$ (0.076) suggests intermixing among Tunisian goats. Genetic distance values range from 0.319 to 0.985.

## 1 Introduction

Goats (*Capra hircus*) have been important to human society for thousands of years, giving milk, meat, fiber, and skin (Mason, 1984). Their ability to adapt to a variety of environmental situations makes them an important resource in many agricultural systems, especially in underdeveloped countries where they are frequently used for subsistence farming (Devendra and Burns, 1983). Goats were among the first domesticated animals, and they are an important source of meat, milk, fiber, and hides in many cultures around the world (Lohani and Bhandari, 2021; Shrestha and Fahmy, 2005). Local goat populations are especially prominent in areas where agriculture and livestock are the main economic activities. The northwest of Tunisia is predominantly home to indigenous goat populations, with the Arbi (local) goat being the most prevalent breed. This breed has evolved well in relation to difficult climatic circumstances and large agricultural systems, demonstrating excellent tolerance to feed shortages and illness (Rekik et al., 2016; Ben Salem et al., 2018). There may also be some Saanen and Alpine crossbreeds since genetic improvement efforts have periodically introduced these breeds to increase milk output (M'sadak et al., 2020). These populations frequently contain unique genetic features tailored to local environmental conditions, making them essential for breeding efforts aiming to increase disease resistance and production (FAO, 2007). Furthermore, understanding the genetic structure of these populations aids in identifying unique genetic groups and their evolutionary ties, which is critical for genetic resource conservation (Luikart et al., 1999). Effective conservation and breeding initiatives require an understanding of the structure and genetic diversity of local goat populations (Kichamu et al., 2024). The genetic diversity within a population is a key indicator of its health and adaptability (Monau et al., 2020). Higher genetic diversity often correlates with greater resilience to diseases and environmental changes (Frankham, 2005). Conversely, low genetic diversity can lead to inbreeding depression, reducing fitness and survival rates (Allendorf and Luikart, 2007). Therefore, assessing the genetic diversity of goat populations is imperative for maintaining their viability and productivity (García et al., 2012).

Previous studies on the genetic diversity of goat populations have employed various molecular markers such as microsatellites, mitochondrial DNA, and single nucleotide polymorphisms (SNPs). These markers have been instrumental in revealing goats' genetic variations and population structures in different regions (Taberlet et al., 2008). For instance, studies using microsatellite markers have shown significant genetic differentiation among goat populations in the Mediterranean region, reflecting their historical and geographical separation (Ajmone-Marsan et al., 2001). Microsatellites and single nucleotide polymorphisms (SNPs) are widely utilized markers because of their high variability and informativeness (Tsunoda et al., 2010). Hussain et al. (2016) used microsatellite markers to identify significant genetic heterogeneity among Pakistani goat breeds, underlining the impact of geographic constraints and breeding techniques on genetic diversity. Similarly, Zenger et al. (2007) employed SNP markers to investigate the genetic structure of Australian goat populations, identifying unique genetic clusters associated with various management techniques. Therefore, the purpose of this research work is to look at the genetic diversity and population structure of local goat populations, which will provide important insights for the long-term management and enhancement of these valuable animals.

## 2 Materials and methods

### 2.1 Sampling procedure

**Figure 1:**
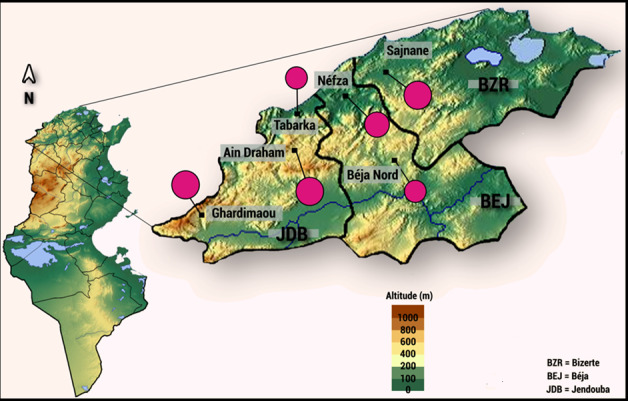
The geographical locations of local goat populations.

A total of 128 indigenous goats were systematically randomly sampled from the six sites in the northwestern region of Tunisia: Sejnane ( n=20 ), Nefza ( n=27 ), Béja Nord ( n=18 ), Ghardimaou ( n=20 ), Ain Draham ( n=20 ), and Tabarka ( n=23 ) (Fig. 1).

The study was carried out with blood samples being collected sterilely from adult animals by means of a puncture to the jugular vein using a vacuum sampling system. After shaving and disinfecting the sampling site, the animal's head was lifted to an angle of around 30°, and pressure was applied to the neck with the finger to swell the vein (Smith, 2010). Blood sampling was performed using a 20 G needle with two bevels: one to puncture the vein and the other to be screwed onto the sampling body to pierce the tube cap. Once the bevel was inserted into the jugular vein (at a 30° angle) and the sampling body was immobilized, the tube was pushed to the bottom of the sampling body so that blood flow could begin. The blood was collected in tubes containing ethylenediaminetetraacetic (EDTA), an anticoagulant that preserves the blood for long periods. After drawing the desired volume of blood, the tube and needle were successively removed, and pressure was applied to stop the bleeding. The tubes were immediately and gently mixed by successive and complete inversion.

### 2.2 DNA extraction and PCR reaction

Blood samples were collected in EDTA tubes and frozen at -20 °C. Genomic DNA extraction was done using the GF-1 Blood DNA Extraction Kit, which employs glass filter membrane technology for isolating and purifying genomic DNA from 200 µL of whole blood. The procedure involves five steps: (1) cell lysis, (2) DNA precipitation, (3) binding of DNA to the glass filter membrane, (4) membrane washing, and (5) DNA elution from the membrane. After bringing blood samples to room temperature (15 to 25 °C), they are lysed with Proteinase K and a lysis buffer, Buffer BB. Ethanol is added to the lysate to promote DNA precipitation and binding to a silica filter membrane in a centrifuge column. The genomic DNA binds to the membrane, while the lysate passes through. Contaminants are removed with two ethanol-based washes (wash buffer 1 and wash buffer 2). Finally, the genomic DNA is eluted from the membrane and collected using the elution buffer.

PCR is carried out in PCR tubes with a reaction mixture that includes 3 µL (50 ng) of template DNA, 0.5 µL of forward primers, 0.5 µL of reverse primers, 3.5 µL of H_2_O, and 12.5 µL of master mix (see Table 2 for composition). The process uses a GeneAmp PCR 9700 thermal cycler programmed to run the following cycles: an initial denaturation at 95 °C, followed by 35 cycles of 30 s at 95 °C for denaturation, 1 min at the primers' annealing temperature for annealing, and 1 min at 72 °C for extension. The final step is a 10 min extension at 72 °C after the last amplification cycle (Mburu and Hanotte, 2005). For this study, seven microsatellite loci labeled with 6-FAM (6-carboxyfluorescein) fluorescence, detailed in Table 1, were selected to analyze the genetic variability of goat populations across six sites. These markers were chosen based on microsatellites used in other research projects and those recommended by FAO (2011) for investigating genetic diversity in goats.

**Table 1:** The microsatellite markers.

Locus	Chromosome	Size of the allele (pb)	Primer sequences (5 ${}^{\prime }\to 3^{\prime }$ )	References
CSRD247	OAR 14	210–260	F: GGACTTGCCAGAACTCTGCAAT R: CACTGTGGTTTGTATTAGTCAGG	Tefiel et al. (2018), Naqvi et al. (2017)
ETH10	CHI 5	190–220	F: GTTCAGGACTGGCCCTGCTAACA R: CCTCCAGCCCACTTTCTCTTCTC	Shanthalatha et al. (2019), Al-Atiyat (2017)
ILSTS029	BTA 3	135–185	F: TGTTTTGATGGAACACAG R: TGGATTTAGACCAGGGTTGG	Naqvi et al. (2017), Al-Atiyat (2017), Aljumaah et al. (2015)
INRA063	CHI 18	145–195	F: GACCACAAAGGGATTTGCACAAGC R: AAACCACAGAAATGCTTGGAAG	Tefiel et al. (2018), Al-Atiyat (2017), Elbeltagy et al. (2016)
MAF065	OAR 15	100–160	F: AAAGGCCAGAGTATGCAATTAGGAG R: CCACTCCTCCTGAGAATATAACATG	Elbeltagy et al. (2016), Meutchieye et al. (2014), Wei et al. (2014)
MCM527	OAR 5	155–195	F: GTCCATTGCCTCAAATCAATTC R: AAACCACTTGACTACTCCCCAA	Shanthalatha et al. (2019), Tefiel et al. (2018), Naqvi et al. (2017)
SRCRSP3	CHI 10	95–135	F: CGGGGATCTGTTCTATGAAC R: TGATTAGCTGGCTGAATGTCC	Naqvi et al. (2017), Aljumaah et al. (2015), Meutchieye et al. (2014)

After the PCR reaction, a denaturation mix is prepared with 8 µL of Hi-Di formamide (which increases PCR specificity) and 0.3 µL of GeneScan 500 LIZ (a size marker). The amount of each ingredient is scaled up according to the number of samples. Then, 8.3 µL of the denaturation mix is distributed into the plate wells based on the number of samples, and 2 µL of PCR product is added. The mixture undergoes denaturation at 95 °C and a preservation cycle at 4 °C for 3 min in a thermal cycler. After denaturation, the plate is placed in the sequencer for capillary electrophoresis. The fragment lengths of the PCR products were estimated with the GeneMapper Software 6.0 (Applied Biosystems). They were then used to construct a genotypic dataset for statistical analyses.

#### 2.2.1 Genetic diversity assessment

The allele richness, rate of polymorphic loci, allele frequencies, private alleles, heterozygosity rates ( H ), fixation index ( $F_{\mathrm{IS}}$ ), genetic differentiation ( $F_{\mathrm{ST}}$ ), and genetic distance between populations (Nei, 1987), as well as the factorial correspondence analysis of populations, were calculated using GENETIX 4.02 software (Belkhir et al., 2001). The STRUCTURE 2.3.3 program (Pritchard et al., 2000) was employed to analyze the genetic structure of our populations. This program infers the number of populations into which the analyzed genotypes can be divided. It estimates the natural logarithm of the probability that a given genotype X belongs to a particular population K : log Pr ( X|K ) given. This ensures that the groups are as representative as possible of samples from a single population. The program assumes that there are K populations with an unknown gene frequency distribution at each locus pkl for k=1…K populations and l=1…L loci contributing to the target population's gene pool. Alleles at each locus are independently sampled for each individual based on the proportion qi of its genotype in a given population. STRUCTURE uses a Markov chain Monte Carlo method to separately estimate the posterior probability distribution of each parameter (particularly qi and qkl) integrated over all other parameters. All analyses used a burn-in period of 100 000 iterations and a data collection period of 100 000 iterations.

## 3 Results and discussions

### 3.1 Intra-population genetic diversity

#### 3.1.1 Allelic richness

Table 2 shows the total number of alleles ( $N_{a}$ ) calculated for each goat population. The seven loci amplified showed complete genetic polymorphism (100 % polymorphism), with allele numbers per locus ranging from 4 (ETH10, MCM527, SRCRSP3) to 13 (INRA063, MCM527).

The total allele counts are 62, 63, 48, 67, 44, and 58 for the populations of Sajnane, Néfza, Béja Nord, Ain Draham, Tabarka, and Ghardimaou, respectively. Allelic richness is influenced by sample size as more individuals increase the likelihood of finding new alleles (Foulley and Ollivier, 2006). According to Table 2, the average number of alleles per locus in the six populations ranges from 6.285 in Tabarka to 9.571 in Ain Draham. This suggests that the Ain Draham population has the highest genetic polymorphism, likely due to its larger herd size. The overall average number of alleles per locus is 8143, indicating considerable genetic diversity. This diversity is evident between markers and populations. Other goat breeds analyzed with similar microsatellite markers also show this genetic variability. The average number of alleles in the six local goat populations is higher than that in China's indigenous goat populations (average of 6.31 alleles, Wei et al., 2014) but lower than that in Algeria's local goat population (average of 14 847 alleles, Tefiel et al., 2018). Nafti et al. (2016) found that the average number of alleles per locus in southern Tunisia's local goat populations ranges from 3.87 in El Hamma to 4.58 in Bechni, which is similar to the Medenine region's populations, where it ranges from 2.83 in Ben Guerdane to 4 in Sidi Makhlouf in the south of Tunisia (Aloui, 2019).

**Table 2:** Total number of alleles ( $N_{t}$ ) and average number of alleles ( $N_{a}$ ).

Locus		Populations						$N_{T}$ /Locus	$N_{a}$ /Pop_t_	Total
		Sajnane	Néfza	Béja Nord	Ain Draham	Tabarka	Ghardimaou			
	N	10	8	6	10	6	10			
CSRD247		10	10	6	9	7	6	18	–	–
ETH10		4	9	7	11	7	11	18	–	–
ILSTS029		9	8	11	10	7	11	19	–	–
INRA063		8	7	6	13	4	9	15	–	–
MAF065		12	10	7	10	7	10	19	–	–
MCM527		13	12	4	9	7	7	22	–	–
SRCRSP3		6	7	7	5	5	4	12	–	–
$N_{t}$ /Pop		62	63	48	67	44	58	–	–	–
$N_{a}$ /Pop_t_		8857	9	6857	9571	6285	8285	–	8.143	–
Number of private alleles		4	9	0	9	4	6	–	–	32
Percentage of private alleles/Pop		12 500	28 125	0	28 125	12 500	18 750			100 %

### 3.2 Polymorphic loci rate

The proportion of polymorphic microsatellites (those with multiple alleles at a given locus among all studied microsatellites) is calculated as follows:

1 $$P=\frac{\text{number of polymorphic genes}}{\text{total number of studied genes}}.$$

In this study, fragment length datasets were created by extracting DNA from samples and amplifying particular SNP markers with polymerase chain reaction (PCR). The fragment lengths (in base pairs) of each sample were recorded to create the dataset. The dataset contains important genetic diversity measures such as allele frequencies, observable heterozygosity ( $H_{O}$ ), anticipated heterozygosity ( $H_{E}$ ), and genetic differentiation ( $F_{\mathrm{ST}}$ ). Table 3, in columns A through V, shows the relative frequencies of each detected allele at a given locus. All markers showed significant genetic polymorphism, averaging 18 different alleles. The genotyping analysis of the northwestern local goat populations revealed that the MCM527 marker was the most polymorphic, with 22 alleles. In contrast, the SRCRSP3 marker was the least polymorphic, with 12 alleles (Table 3). All loci were found to be 100 % polymorphic ( P=100 %) at the 95 % threshold across the six populations, demonstrating the effectiveness of the markers in studying genetic diversity.

### 3.3 Allele frequencies and private alleles

The genotyping of microsatellites revealed the individual genotypes, detecting a total of 123 alleles and 233 different genotypes across seven loci in six populations. This indicates significant polymorphism within the local goat population. Table 3 illustrates this genetic diversity, showing allele distributions and frequencies at each locus for each population. The allele distribution is quite consistent across populations, with frequent alleles generally being the same. For example, allele K is often the most common across various loci in all studied populations. Allele frequencies for each locus range from 0.050 for several alleles to 0.450 for allele K of SRCRSP3 in the Ain Draham population. By examining these frequencies, specific or private alleles unique to a population can be identified. The distribution of these alleles highlights the genetic variability among different populations. The study identified 32 specific alleles out of 123 alleles, with frequencies from 5 % for most alleles ( 15/32 ) to 15 % for allele SRCRSP3-112 in the Sajnane population. Given these findings, we have examined the results for each microsatellite locus, which will be presented in our analysis. The observed genetic diversity and allele distribution are comparable with previous studies on goat populations. For example, studies on Swiss goat breeds have found high levels of polymorphism and significant genetic variability, as well as specific alleles that are unique to each breed (Glowatzki-Mullis et al., 2008). Similarly, research on Turkish goat populations has indicated significant genetic diversity, with a large number of alleles being found at multiple loci (Ünal et al., 2021). The prevalence of private alleles and high polymorphism levels in the analyzed populations indicates a large genetic pool that could be used for selective breeding programs aiming to improve specific traits (Mahapatra et al., 2018). This genetic variety also demonstrates the populations' abilities to adapt to environmental changes and withstand sickness, contributing to their long-term viability (Bijlsma and Loeschcke, 2012).

**Table 3:** Allelic frequencies of abundant and deprived alleles across different populations (letters A to V represent the alleles).

Loci	Population	$N_{a}$	Allelic frequencies																					
			A	B	C	D	E	F	G	H	I	G	K	L	M	N	O	P	Q	R	S	T	U	V
CSRD247	Sajnane	10	0	0	0	0	0.050	0.050	0.050	0.050	0.050	0.050	0	0.200	0	0	0	0.300	0.150	0.050				
	Néfza	10	0	0.063	0	0	0	0	0.063	0	0.063	0.063	0.250	0.125	0	0	0.063	0.188	0.063	0.063				
	Béja Nord	6	0.417	0.083	0	0	0	0	0	0	0	0	0.083	0.083	0	0	0.083	0	0.250	0				
	Ain Draham	9	0.100	0	0	0.050	0.150	0	0	0.200	0	0	0.100	0.150	0	0.050	0	0.100	0.100	0				
	Tabarka	7	0.160	0	0.083	0.083	0	0	0	0	0	0.083	0.333	0	0	0	0	0.083	0.167	0				
	Ghardimaou	6	0	0	0	0.200	0.200	0	0	0	0	0.050	0	0	0.050	0	0	0.050	0.450	0				
Size of the allele (pb)			220	222	223	228	229	230	232	233	234	235	237	239	240	241	242	243	245	246				
ETH10	Sajnane	4	0	0	0.400	0.100	0	0	0	0	0	0	0	0.400	0	0.100	0	0	0	0				
	Néfza	9	0.063	0.063	0.188	0.063	0	0	0	0	0.063	0	0.188	0.063	0.250	0	0	0.063	0	0				
	Béja Nord	7	0	0	0	0.167	0	0	0	0.083	0.083	0	0.083	0	0.333	0.167	0.083	0	0	0				
	Ain Draham	11	0.100	0.050	0.150	0.150	0	0.050	0	0	0	0.050	0.050	0.100	0.100	0.150	0	0	0	0.050				
	Tabarka	7	0	0	0.083	0.083	0	0	0	0	0	0	0.167	0.083	0.417	0	0.083	0.083	0	0				
	Ghardimaou	11	0	0	0.050	0.050	0.100	0	0.100	0.050	0	0.100	0.200	0	0.150	0	0.100	0.050	0.050	0				
Size of the allele (pb)			195	196	197	198	200	202	204	206	207	209	210	211	212	213	214	215	216	218				
ILSTS029	Sajnane	9	0.050	0.050	0	0.050	0.100	0	0.100	0	0.050	0	0.250	0.250	0.100	0	0	0	0	0	0			
	Néfza	8	0	0.063	0	0.063	0.188	0	0.188	0	0	0	0.250	0.125	0	0.063	0	0.063	0	0	0			
	Béja Nord	11	0.083	0.083	0	0.083	0.083	0	0	0	0.083	0.083	0.167	0	0.083	0	0	0	0.083	0.083	0.083			
	Ain Draham	10	0	0.050	0	0	0	0.050	0	0.100	0	0.100	0.250	0	0.200	0.050	0.050	0.050	0.100	0	0			
	Tabarka	7	0	0	0	0.083	0.250	0	0.083	0.083	0	0	0.167	0	0.250	0	0	0.083	0	0	0			
	Ghardimaou	11	0	0.050	0.050	0	0.150	0.050	0.150	0	0	0	0.250	0.050	0.100	0	0	0	0.050	0.050	0.050			
Size of the allele (pb)			150	151	153	154	155	156	157	158	160	162	163	164	165	167	168	169	170	171	175			
INRA063	Sajnane	8	0	0	0.050	0.250	0.050	0.050	0	0.050	0	0.100	0	0.150	0	0.300	0							
	Néfza	7	0	0	0	0.063	0.063	0.063	0.063	0	0	0.250	0.125	0	0	0.375	0							
	Béja Nord	6	0	0	0	0	0	0	0	0.167	0	0.083	0.083	0	0.333	0.083	0.250							
	Ain Draham	13	0.050	0.050	0	0.050	0.100	0.050	0.100	0.050	0.050	0.100	0.100	0.100	0.050	0.150	0							
	Tabarka	4	0	0	0	0	0	0	0	0.083	0	0.417	0	0	0.167	0.333	0							
	Ghardimaou	9	0	0	0.050	0.050	0	0	0.200	0	0.050	0.150	0.150	0.100	0	0.200	0.050							
Size of the allele (pb)			166	167	168	170	172	174	175	176	177	178	179	181	182	183	185							
MAF065	Sajnane	12	0	0.200	0.100	0.050	0.050	0.150	0.050	0	0.100	0	0.050	0	0.050	0	0	0.100	0.050	0.050	0			
	Néfza	10	0	0	0	0	0	0.125	0.313	0.063	0.063	0.125	0.063	0.063	0.063	0.063	0	0	0.063	0	0			
	Béja Nord	7	0.083	0	0	0	0.250	0	0.167	0	0.167	0	0.083	0.083	0	0	0	0	0	0.167	0			
	Ain Draham	10	0.050	0.100	0	0.050	0.100	0.150	0	0	0	0	0.150	0	0	0.050	0	0.200	0	0.100	0.050			
	Tabarka	7	0.083	0	0.333	0	0	0	0.250	0	0.083	0.083	0	0	0	0.083	0.083	0	0	0	0			
	Ghardimaou	10	0	0.150	0.100	0.050	0.050	0.250	0.050	0	0	0.050	0.050	0	0	0.100	0	0.150	0	0	0			
Size of the allele (pb)			114	117	118	119	120	121	122	124	126	127	129	130	131	132	133	134	135	136	150			
MCM527	Sajnane	13	0	0.150	0	0	0.050	0	0.050	0.050	0.100	0.050	0.050	0.100	0.100	0	0.050	0.050	0	0.150	0.050	0	0	0
	Néfza	12	0.063	0.063	0.063	0.063	0	0	0	0.063	0.125	0	0.063	0.063	0	0.125	0.188	0.063	0	0	0	0	0	0.063
	Béja Nord	4	0	0.333	0	0	0	0	0.083	0	0.417	0	0	0	0	0	0	0.167	0	0	0	0	0	0
	Ain Draham	9	0	0	0	0	0	0	0	0	0.100	0	0.050	0.050	0.300	0	0.050	0.050	0.050	0.250	0	0	0.100	0
	Tabarka	7	0	0.083	0	0	0	0	0.083	0	0.250	0	0	0.167	0	0	0	0.250	0	0.083	0	0.083	0	0
	Ghardimaou	7	0	0	0	0	0	0.050	0	0	0.250	0	0	0.150	0.050	0	0	0.150	0	0.200	0.150	0	0	0
Size of the allele(pb)			139	143	145	147	149	151	152	153	154	156	157	160	162	163	164	165	166	167	169	171	173	175
SRCRSP3	Sajnane	6	0	0.050	0	0.050	0	0.150	0.300	0	0.150	0	0.300	0										
	Néfza	7	0.063	0.188	0	0	0.063	0	0.250	0	0.063	0.063	0.313	0										
	Béja Nord	7	0	0.250	0	0.083	0	0	0.167	0.083	0.083	0	0.250	0.083										
	Ain Draham	5	0	0.100	0	0	0	0	0.350	0.050	0	0	0.450	0.050										
	Tabarka	5	0	0	0.083	0	0	0	0.333	0.083	0	0	0.417	0.083										
	Ghardimaou	4	0	0	0	0	0	0	0.400	0.100	0	0	0.400	0.100										
Size of the allele (pb)			100	105	109	110	111	112	114	116	117	119	120	122										

#### 3.3.1 Locus CSRD247

**Figure 2:**
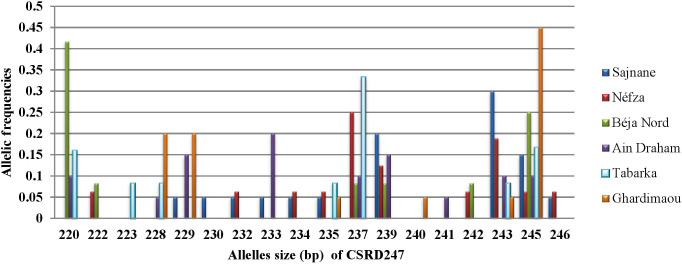
Allelic frequencies for locus CSRD247.

The analysis of the CSRD247 genetic locus, as illustrated in Fig. 2, revealed that this locus produced 18 different alleles across six populations, with allele sizes ranging from 220 to 246 bp in the Béja Nord and Ghardimaou populations, respectively. The allele with a size of 245 bp was present in every population, but its frequency varied, being most common in Ghardimaou, where it was found in 45 % of the goats. Additionally, four unique alleles (private alleles) were identified. The allele found in the Tabarka population had a frequency of 0.083, which is relatively higher compared to the frequencies of private alleles in other populations. The locus CSRD247 exhibits significant genetic diversity among the studied populations. Additionally, the presence of private alleles unique to certain populations underscores their genetic distinctiveness, with Tabarka showing a particularly high frequency of such private alleles.

#### 3.3.2 Locus ETH10

**Figure 3:**
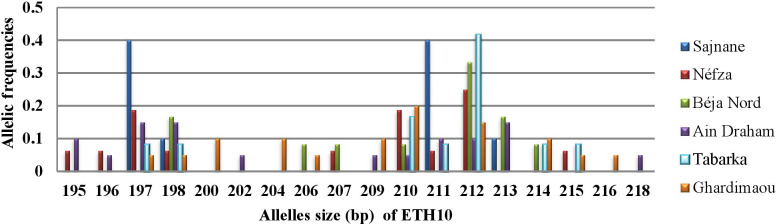
Allelic frequencies of the ETH10 locus.

The analysis of the amplicons for this locus reveals the presence of 18 alleles, with sizes ranging from 195 to 218 bp. The histograms in Fig. 3 show a heterogeneous distribution of alleles across different populations. Major alleles were observed at sizes of 197, 210, 211, and 212 bp, with the Sajnane population showing a higher frequency for the 197 and 211 bp alleles. Unique or private alleles were found in two populations: that of Ain Draham, which had two distinct alleles with a frequency of 0.050, and that of Ghardimaou, which had three private alleles with varying frequencies. This indicates genetic diversity and population-specific allele distributions.

#### 3.3.3 Locus ILSTS029

**Figure 4:**
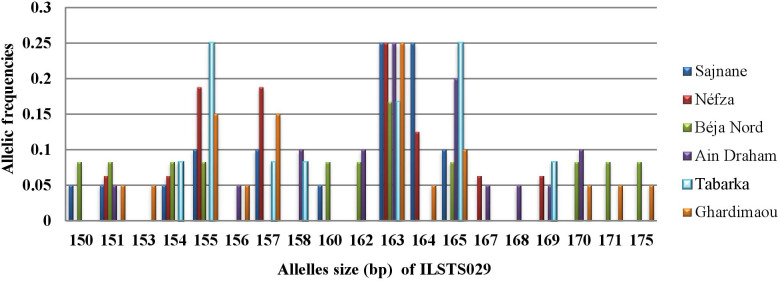
Allelic frequencies for locus ILSTS029.

Locus ILSTS029 exhibited a total of 19 alleles with sizes ranging from 150 to 175 bp. The major alleles were sized at 155 and 163 bp in five populations (Sajnane, Néfza, Béja Nord, Ain Draham, and Ghardimaou) (Fig. 4). However, the Sajnane population also had a distinct major allele sized at 164 bp. Additionally, the Tabarka population showed two major alleles sized at 155 and 165 bp, each with a frequency of 0.250. There were also two private alleles identified, one sized at 153 bp and another sized at 168 bp, both with a frequency of 0.050, indicating specific genetic variations unique to certain populations.

#### 3.3.4 Locus INRA063

**Figure 5:**
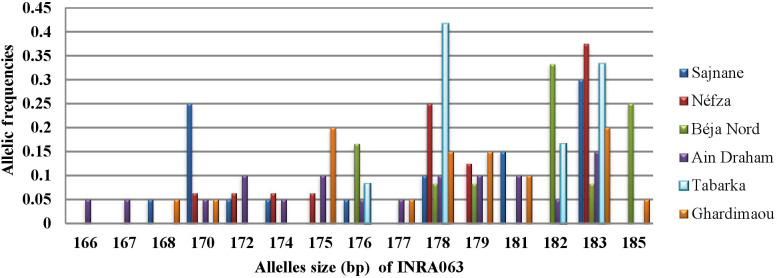
Allelic frequencies for locus INRA063.

For locus INRA063, allele sizes ranged from 166 to 185 bp, resulting in a total of 15 different alleles. Figure 5 shows the presence of four major alleles with sizes of 175, 178, 182, and 183 bp. In the Ghardimaou population, the alleles, each sized at 175 bp and 183 bp, had similar frequencies of approximately 0.200. Additionally, two private alleles were identified in the Ain Draham population, with sizes of 166 and 167 bp, each having a frequency of 0.050, indicating a unique regional genetic signature.

#### 3.3.5 Locus MAF065

**Figure 6:**
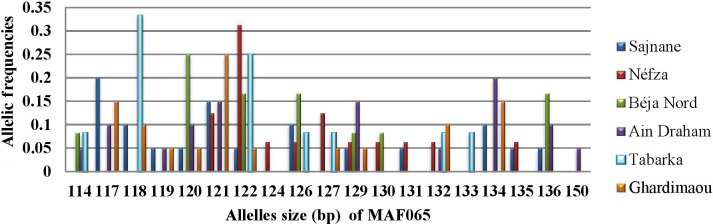
Allelic frequencies for locus MAF065.

Locus MAF065 revealed the presence of 19 alleles across the six populations, with sizes ranging from 114 to 150 bp. Figure 6 illustrates the genotyping profiles of individuals. The frequencies of the alleles varied among sites. Notably, the 118 bp allele had a relatively high frequency in the goats from Tabarka. The populations of Néfza, Tabarka, and Ain Draham stand out compared to the others due to having unique private alleles sized at 124, 133, and 150 bp, respectively, highlighting distinctive genetic features in these populations.

#### 3.3.6 Locus MCM527

**Figure 7:**
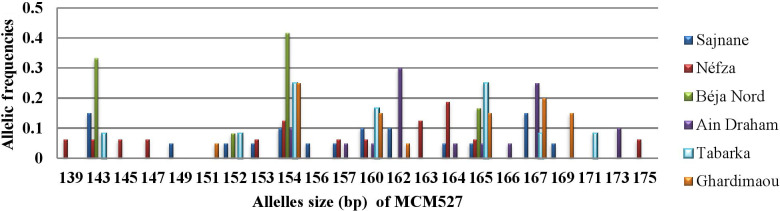
Allelic frequencies for locus MCM527.

The analysis of the genotyping profiles in the goat population revealed that locus MCM527 is the most polymorphic, with a total of 22 alleles identified (Fig. 7). The results showed the presence of new alleles outside the size ranges of [165–187], defined by FAO (2011), and [155–195], defined by Mburu and Hanotte (2005). Notably, the major alleles in the Béja Nord and Néfza populations are outside these size intervals. Capillary electrophoresis may yield inaccurate results for alleles outside the established size range, making sequencing recommended for accurate allele designation and exact base pair length. The observed polymorphism and detection of new alleles may explain these findings. Additionally, the number of private alleles for this marker is significantly higher compared to other loci, with a notably different distribution pattern. Among the six local goat populations, alleles 139, 145, 147, 163, and 175 were found exclusively in Néfza goats, with a frequency of 0.063 for the majority, suggesting a higher overall polymorphism in this population. Other private alleles with varying frequencies are prominent in each population, except for Béja Nord.

#### 3.3.7 Locus SRCRSP3

**Figure 8:**
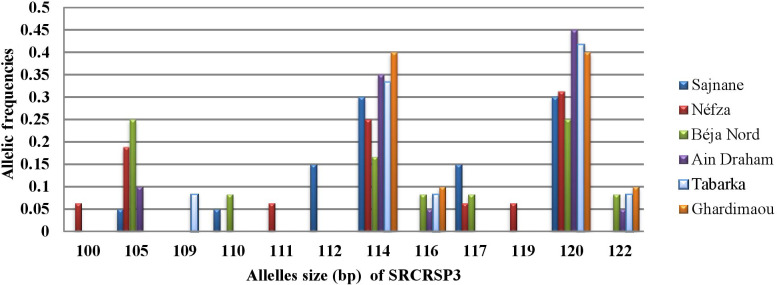
Allelic frequencies for locus SRCRSP3.

Figure 8 shows the distribution of allele frequencies for the microsatellite SRCRSP3 across the six studied populations. This locus is the least polymorphic, with only 12 alleles observed. Alleles sized at 114 and 120 bp are present in all populations, with equal allele frequencies of 0.300 and 0.400 in the Sajnane and Ghardimaou populations. The 120 bp allele is also the major allele in the Tabarka and Ain Draham populations, with frequencies of 0.417 and 0.450, respectively. There are five private alleles with frequencies ranging from 0.063 to 0.150, indicating some unique genetic variation in different populations. The private allele associated with the Sajnane region, sized at 112 bp, has the highest frequency observed for all loci at 0.150, highlighting its significant presence in that region.

### 3.4 Heterozygosity rate ( H )

We determined the observed ( $H_{O}$ ) and expected ( $H_{E}$ ) heterozygosity rates for each population under the assumption of Hardy–Weinberg equilibrium (Table 4). To evaluate genetic polymorphism, we compared these heterozygosity rates. A higher observed heterozygosity rate compared to the expected rate indicates an excess of heterozygotes in the population. Our findings reveal that all loci studied are in Hardy–Weinberg equilibrium, except for the marker ILSTS029, which shows a heterozygosity deficit in five populations (Table 5). Similar deficits were noted for the loci ETH10 and MCM527 in the goat populations of Ghardimaou and Néfza, respectively. These results are consistent with previous research on animal genetic diversity. Studies on Swiss goat populations have found comparable heterozygosity patterns, with most loci being in Hardy–Weinberg equilibrium and with occasional deficits indicating possible inbreeding or selection pressures (Glowatzki-Mullis et al., 2008). Research on Turkish goats indicated similar trends, with most loci matching Hardy–Weinberg expectations and with certain loci displaying heterozygosity deficits (Kaya and Yildiz, 2008). Furthermore, research on cow populations has shown that deviations from the Hardy–Weinberg equilibrium at certain loci might reveal underlying genetic processes such as selection or population substructures (Kuehn et al., 2011). These differences are critical for understanding the genetic dynamics of populations and adopting successful breeding and conservation methods.

**Table 4:** Observed ( $H_{O}$ ) and expected ( $H_{E}$ ) heterozygosity rates by population for the seven microsatellites.

Populations	Sajnane		Néfza		Béja Nord		Ain Draham		Tabarka		Ghardimaou	
Locus	$H_{O}$	$H_{E}$	$H_{O}$	$H_{E}$	$H_{O}$	$H_{E}$	$H_{O}$	$H_{E}$	$H_{O}$	$H_{E}$	$H_{O}$	$H_{E}$
CSRD247	0.900	0.830	1.000	0.859	0.833	0.736	1.000	0.870	0.833	0.806	1.000	0.710
ETH10	1.000	0.660	1.000	0.844	1.000	0.806	1.000	0.890	0.833	0.764	0.700	0.885
ILSTS029	0.600	0.835	0.750	0.836	0.833	0.903	0.700	0.855	1.000	0.819	0.800	0.865
INRA063	1.000	0.805	1.000	0.766	1.000	0.778	1.000	0.910	1.000	0.681	1.000	0.855
MAF065	1.000	0.890	1.000	0.844	1.000	0.833	1.000	0.875	1.000	0.792	1.000	0.860
MCM527	1.000	0.905	0.875	0.898	1.000	0.681	0.900	0.815	1.000	0.819	1.000	0.825
SRCRSP3	1.000	0.770	1.000	0.789	1.000	0.819	1.000	0.660	1.000	0.694	1.000	0.660
Multi-locus	0.929	0.814	0.946	0.834	0.952	0.794	0.943	0.839	0.952	0.768	0.929	0.809

The observed heterozygosity values for goat populations range from 0.929 in Sajnane and Ghardimaou to 0.952 in Béja Nord and Tabarka. These values are higher than those reported by Elbeltagy et al. (2016), who used 14 microsatellite markers to characterize North African goat populations (Zaraibi, Baladi, Saidi, Barki, and Shami) and found heterozygosity rates of between 0.640 and 0.710 for Zaraibi and Shami. Similarly, Al-Atiyat (2017) reported lower expected heterozygosity rates (0.699 to 0.749) for 10 goat breeds from the Middle East and the Horn of Africa using 17 microsatellite markers. In contrast, our study's observed heterozygosity value (0.929) is notably high compared to other studies, such as that of Naqvi et al. (2017), who found HO values of 0.540, 0.575, 0.612, 0.550, and 0.580 for Beetal, Kaghani, Teddy, Nachi, and Pahari goats from Pakistan. For the overall population, the expected heterozygosity (0.809) is lower than the observed heterozygosity (0.942), indicating an excess of heterozygosity in the local goat population in northwestern Tunisia. This value is similar to that of Brazilian goats (0.717) (Araújo et al., 2010), lower than that of Algerian goats (0.94) (Tefiel et al., 2018), and higher than that of Chinese goats (0.644) (Wei et al., 2014) and Yunnan goats (0.599) (Guang-Xin et al., 2019). Heterozygosity deficits in goat populations can result from several factors, including inbreeding, genetic drift in small or isolated populations, selection pressure, low mutation rates, and non-random mating.

**Table 5:** Observed ( $H_{O}$ ) and expected ( $H_{E}$ ) heterozygosity rates by microsatellite marker.

Locus	$H_{O}$	$H_{E}$
CSRD247	0.928	0.802
ETH10	0.922	0.808
ILSTS029	0.781	0.852
INRA063	1.000	0.799
MAF065	1.000	0.849
MCM527	0.963	0.824
SRCRSP3	1.000	0.732
Mean	0.942	0.809

### 3.5 Fixation index $F_{\mathrm{IS}}$

The inbreeding coefficients (FIS) for each of the goat populations studied are presented in Table 6. The FIS index informs us about the deviation in terms of observed heterozygosity in an individual compared to the expected heterozygosity under random mating (Hardy–Weinberg model). This index also provides information on the degree of inbreeding among individuals resulting from matings between closely related individuals within the studied goat populations. The overall inbreeding coefficient, FIS, is -0.177 . This negative value indicates an overall excess of heterozygotes, suggesting the absence of inbreeding within the six populations as these are the result of a breeding regime between different individuals. The six populations show negative fixation indices (FIS) that vary between -0.248 for the Tabarka population and -0.136 for the Ain Draham population. Despite these negative values, we observed positive indices for the locus ILSTS029, indicating a heterozygote deficit in five populations. It should be noted that some populations show significant excesses of homozygotes compared to Hardy–Weinberg equilibrium proportions, namely the Ghardimaou and Néfza goats for the loci ETH10 and MCM527. However, other populations (Sajnane, Ain Draham, and Ghardimaou) have significant excesses of heterozygotes compared to Hardy–Weinberg equilibrium proportions at certain loci where the FIS value reaches -0.515 . The FIS values found in this study are lower than those reported in a study on two Moroccan goat breeds (Hilal et al., 2016) and those previously found in Chinese populations (Wei et al., 2014), indicating an excess of heterozygotes in our total population. The findings indicate that the studied goat populations generally exhibit an excess of heterozygotes, suggesting a lack of inbreeding. This is evidenced by the overall negative FIS value of -0.177 . The variation in FIS values among different populations and loci highlights the genetic diversity within these populations. Some loci showed significant deviations from the Hardy–Weinberg equilibrium, with certain populations displaying excesses of homozygotes or heterozygotes. Compared to other studies on Moroccan and Chinese goat populations, the studied populations have a higher presence of heterozygotes, indicating better genetic diversity and less inbreeding (Hilal et al., 2016; Wei et al., 2014).

**Table 6:** $F_{\mathrm{IS}}$ values by microsatellite marker and population.

Locus	Sajnane	Néfza	Béja Nord	Ain Draham	Tabarka	Ghardimaou
CSRD247	-0.084	0.164	-0.132	0.149	-0.034	-0.408
ETH10	-0.515	-0.185	-0.241	-0.124	-0.091	0.209
ILSTS029	0.281	0.103	0.077	0.181	-0.220	0.075
INRA063	-0.242	-0.306	-0.286	-0.099	-0.469	-0.170
MAF065	-0.124	-0.185	-0.200	-0.143	-0.263	-0.163
MCM527	-0.105	0.026	-0.469	-0.104	-0.220	-0.212
SRCRSP3	-0.299	-0.267	-0.220	-0.515	-0.440	-0.515
Mean	-0.155	-0.140	-0.210	-0.136	-0.248	-0.169
Multi-locus	-0.177					

### 3.6 Inter-population genetic diversity

#### 3.6.1 Genetic differentiation ( $F_{\mathrm{ST}}$ )

The genetic differentiation values (FST) for each marker indicate the genetic structure within a total population divided into sub-populations. Out of the seven loci, five have an FST index above 0.05, with the CSRD247 locus showing a relatively high FST of 0.104, while the ILSTS029 and SRCRSP3 loci have lower values of 0.048 and 0.043, respectively (Table 7). The overall FST for the total population is 0.076, which is considered to be moderate and suggests moderate genetic differentiation among the studied populations. This means that only 7.6 % of the total genetic variability is due to differences between populations, while 92.4 % is due to variation within populations. The relatively high FST value for the CSRD247 locus and the lower values for the ILSTS029 and SRCRSP3 loci highlight the variability in genetic differentiation across different loci. The findings from this study align with the results from other research on goat genetic diversity. For instance, studies on European and Middle Eastern goat populations have reported similar $F_{\mathrm{ST}}$ levels of around 7 % (Canon et al., 2006; Colli et al., 2018). Additionally, North African goat populations have shown an FST value of 7.1 %, while Turkish goats have exhibited an $F_{\mathrm{ST}}$ value of 7.5 % (Saitbekova et al., 1999; Kaya and Yildiz, 2008). These consistent $F_{\mathrm{ST}}$ values across different studies highlight the moderate genetic differentiation commonly observed among goat populations, regardless of geographic location. The relatively high $F_{\mathrm{ST}}$ value for the CSRD247 locus suggests greater genetic differentiation at this particular marker, which could be due to selective pressures or genetic drift affecting this locus more significantly than others. On the other hand, the lower $F_{\mathrm{ST}}$ values for the ILSTS029 and SRCRSP3 loci indicate less genetic differentiation, possibly reflecting more conserved regions of the genome (Gupta and Varshney, 2000). Overall, the moderate FST levels observed in this study suggest a genetic structure influenced by random mating, shared ancestry, and commercial interactions between populations (Rousset, 2008; Hedrick, 2005). These factors contribute to maintaining genetic variability within populations while allowing for some degree of differentiation between them (Luo et al., 2012; Gizaw et al., 2013).

**Table 7:** Genetic differentiation ( $F_{\mathrm{ST}}$ ).

Locus	$F_{\mathrm{ST}}$
CSRD247	0.104
ETH10	0.091
ILSTS029	0.048
INRA063	0.086
MAF065	0.081
MCM527	0.082
SRCRSP3	0.043
Multi-locus	0.076

#### 3.6.2 Genetic distances and population dendrograms

The genetic distances between the six goat populations studied, as shown in Table 8, range from 0.319 to 0.985. The smallest genetic distance (0.319) is between the Tabarka and Néfza populations, while the largest (0.985) is between the Sajnane and Béja Nord populations, indicating that the latter are the most genetically distinct. The genetic distance matrix reveals high values, confirming the genetic divergence among the goat populations. These findings are consistent with those of El-Sayed et al. (2017), who reported genetic distances of between 0.380 and 0.967 in Egyptian goats using six genetic markers. Toro and Maki-Tanila (2007) suggested that such genetic divergence is likely to be due to overlapping generations and the mixing of populations from different geographical areas. The findings indicate significant genetic divergence among the six studied goat populations, with genetic distances ranging from 0.319 to 0.985. The smallest distance between the Tabarka and Néfza populations suggests that they are genetically similar, while the largest distance between the Sajnane and Béja Nord populations indicates that they are the most genetically distinct. These high genetic distance values confirm the genetic diversity within the studied populations. Similar findings have been observed in previous research on livestock genetic diversity. For example, research on Swiss goat breeds has revealed significant genetic divergence between geographically isolated populations, emphasizing the effect of geographical isolation on genetic diversity (Glowatzki-Mullis et al., 2008). Studies on Turkish goats have also revealed significant genetic differences between populations, indicating the impact of geographical and historical variables on genetic diversity (Kaya and Yildiz, 2008). Furthermore, research on cow populations has shown that genetic distance is a valid indication of genetic variety, providing useful information for breeding and conservation programs (Loftus et al., 1999). These findings underline the need to preserve genetic variety in animal herds to preserve their long-term viability and adaptation.

**Table 8:** Genetic distance matrix ( D ) for the six populations.

	Sajnane	Néfza	Béja Nord	Ain Draham	Tabarka	Ghardimaou
Sajnane	0.000					
Néfza	0.444	0.000				
Béja Nord	0.985	0.722	0.000			
Ain Draham	0.393	0.566	0.719	0.000		
Tabarka	0.675	0.319	0.491	0.578	0.000	
Ghardimaou	0.534	0.518	0.729	0.356	0.410	0.000

**Figure 9:**
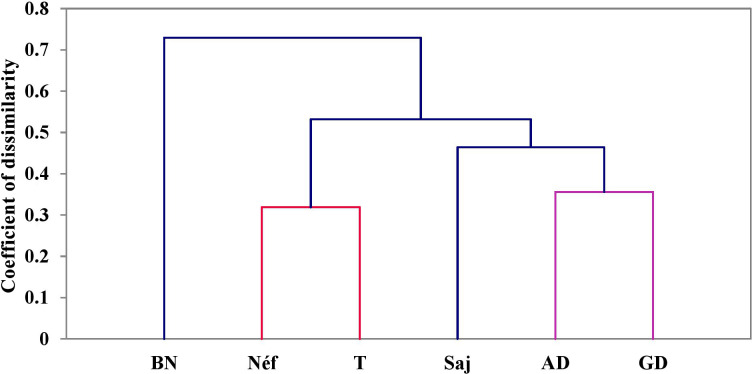
Dendrogram of study populations.

The dendrogram of the populations (Fig. 9), created using the UPGMA (unweighted pair group method with arithmetic mean) method and Nei's (1978) genetic distance calculations, shows four levels of divergence. The Béja Nord population is the first to diverge from the others. The second divergence level groups Ain Draham, Ghardimaou, and Sajnane together and Tabarka and Néfza together. Within this level, Sajnane diverges both genetically ( D=0.356 ) and geographically from Ain Draham and Ghardimaou, forming a separate cluster with a genetic distance of 0.985 from Béja Nord. The final cluster shows the genetic divergence of the closely related Tabarka and Néfza populations, which unite at a genetic distance of 0.319, reflecting their geographical closeness. The findings from the dendrogram analysis indicate four distinct levels of genetic divergence among the studied goat populations. The Béja Nord population is the most genetically distinct, diverging first from the other populations. The second level of divergence groups Ain Draham, Ghardimaou, and Sajnane together and Tabarka and Néfza together. Within this level, Sajnane shows a significant genetic and geographical divergence from Ain Draham and Ghardimaou, forming a separate cluster. The final cluster highlights the close genetic relationship between Tabarka and Néfza, which is also supported by their geographical proximity. These results suggest a complex genetic structure influenced by both genetic and geographical factors. These findings are consistent with previous research on animal genetics, which emphasizes the impact of geographical and environmental factors in producing genetic variety. For example, studies on sheep populations have demonstrated that geographical barriers and differing breeding strategies can result in significant genetic divergence between populations (Ilie et al., 2018; Karsli et al., 2020). Similarly, research on goat populations in various countries has shown that genetic diversity is driven by both natural selection and human-mediated variables including breeding and migration patterns (Visser et al., 2004; Pogorevc et al., 2021).

## 4 Correspondence factor analysis on populations

**Figure 10:**
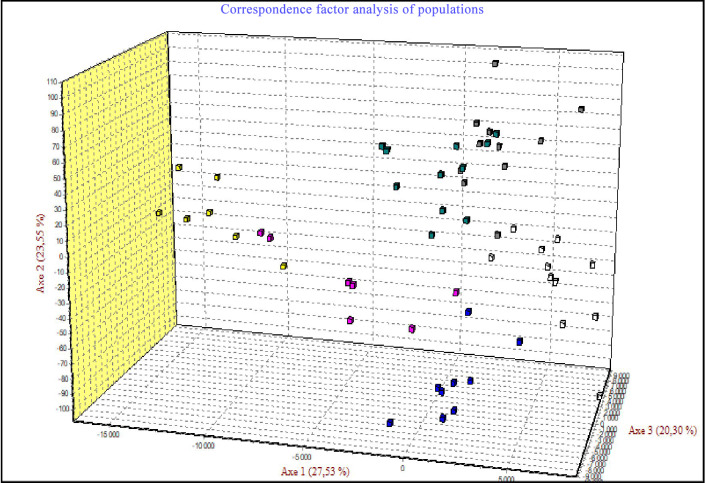
Results of factorial correspondence analysis (FCA) of all six populations for the seven microsatellites studied.

The correspondence factor analysis (CFA) was conducted using the genotyping data of seven microsatellites. The CFA results, shown in Fig. 10, indicate that 71.38 % of the total inertia is divided as follows: 27.53 % for the first axis, 23.55 % for the second axis, and 20.30 % for the third axis. The data analysis along these three axes reveals three distinct groups. The first group includes two populations (Ghardimaou and Ain Draham), with some individuals from the Sajnane population moving towards this group without fully integrating. This clustering suggests a close genetic relationship among these individuals. Additionally, individuals in this group are mainly concentrated at an intermediate level between the three populations, indicating minor genetic differences. In contrast, the second group comprises individuals from the Néfza population, with a few from Tabarka joining this group. This unexpected result might be due to the geographical closeness of these populations. The third group consists solely of individuals from the Béja Nord population, highlighting their genetic divergence. The correspondence factor analysis (CFA) of microsatellite genotyping data reveals three main genetic groups: the first group includes populations from Ghardimaou and Ain Draham, with some individuals from Sajnane moving towards it but not fully integrating, indicating close genetic relationships and minor differences; the second group consists of individuals from Néfza and a few from Tabarka, likely due to geographical proximity influencing genetic similarities; and the third group is solely composed of individuals from Béja Nord, confirming their genetic divergence from the other populations. These results are consistent with previous research on animal genetic diversity. Similar CFA findings have been observed in investigations of Swiss goat populations, where separate genetic groupings were discovered depending on geographical and historical characteristics (Glowatzki-Mullis et al., 2008). Research on Turkish goats found distinct genetic clustering among groups, which was driven by both geographical closeness and historical breeding patterns (Kaya and Yildiz, 2008). Furthermore, CFA research on cow populations has revealed that genetic clustering is frequently associated with geographical and historical factors that influence gene flow and genetic diversity (Kuehn et al., 2011). These studies underscore the necessity of taking into account both genetic data and geographical context when examining genetic linkages among cattle populations.

## 5 Conclusion

The study on the genetic diversity and demographic structure of the indigenous goat population in Tunisia's northwestern region gives important information for improving breeding methods. The study used seven microsatellites to indicate high genetic diversity and obvious population grouping despite geographical dispersion. The study found an average of 18 alleles per marker and 123 alleles across seven loci, confirming significant genetic variability. The greatest observed heterozygosity ( $H_{O}$ ) of 0.952 and the anticipated heterozygosity ( $H_{E}$ ) of 0.942, together with a negative $F_{\mathrm{IS}}$ value of -0.177 , imply an overabundance of heterozygotes and minimal inbreeding. Analysis of molecular variance (AMOVA) studies revealed that 92.4 % of genetic diversity occurred within groups, with an average $F_{\mathrm{ST}}$ of 0.076, indicating significant gene flow between populations. Genetic distance estimates ranging from 0.319 to 0.985 highlight the genetic diversity of these goats. The significance of this study stems from its contribution to understanding the genetic landscape of Tunisian goats, which is critical for conservation and breeding programs aiming to maintain genetic variety and increase productivity. However, the study is hampered by the small number of loci examined, as well as the need for more comprehensive sampling across multiple areas and ecological zones to properly capture the genetic diversity of Tunisia's goat population. Future research should investigate integrating more loci and broadening the geographic breadth of sampling to ensure a more complete picture of genetic diversity. Furthermore, combining genomic data with phenotypic features may provide more detailed insights into the genetic basis of economically significant traits, hence assisting breeding efforts.

## Data Availability

The corresponding authors can provide all raw data upon request.
